# Longitudinal Effects of Mindfulness Combined with Gratitude Touch on Anxiety, Depression, and Stress: A 12-Month Portable EEG-Based Study

**DOI:** 10.3390/brainsci16040425

**Published:** 2026-04-18

**Authors:** Mădălina Sarca, Iuliana-Anamaria Trăilă, Teodora Anghel, Lavinia Bratu, Laura Nussbaum, Ion Papavă, Lavinia Hogea

**Affiliations:** 1 School of Medicine, “Victor Babes” University of Medicine and Pharmacy, 300041 Timisoara, Romania; madalina.sarca@umft.ro (M.S.); iuliana.traila@umft.ro (I.-A.T.); 2 Neuroscience Department, “Victor Babes” University of Medicine and Pharmacy, 300041 Timisoara, Romania; bratu.lavinia@umft.ro (L.B.); nussbaum.laura@umft.ro (L.N.); papava.ion@umft.ro (I.P.); hogea.lavinia@umft.ro (L.H.); 3 Neuropsychology and Behavioral Medicine Center, “Victor Babes” University of Medicine and Pharmacy, 300041 Timisoara, Romania

**Keywords:** mindfulness, gratitude touch, anxiety and depression, stress, electroencephalography (EEG), MUSE EEG, longitudinal study

## Abstract

**Highlights:**

**What are the main findings?**

- A 12-month mindfulness-based intervention combined with a gratitude-focused somatic technique was associated with significant and sustained reductions in depression, anxiety, and perceived stress.
- Longitudinal EEG analyses showed increased alpha power and decreased beta and gamma activity, suggesting time-dependent neurophysiological adaptation.

**What are the implications of the main findings?**

- The findings support the potential of combined cognitive–somatic mindfulness interventions as scalable approaches for long-term mental health improvement.
- Portable EEG may serve as a feasible tool for monitoring dynamic neurophysiological changes associated with psychological interventions in real-world settings.

**Abstract:**

**Background/Objectives**: Mindfulness-based interventions are widely used to reduce psychological distress. Their long-term neurophysiological correlates remain insufficiently characterized. Using a portable Muse InteraXon^®^ EEG device, this study aimed to evaluate (i) the extent to which a 12-month combined mindfulness and gratitude-based intervention reduces anxiety, depression, and perceived stress, and (ii) whether these changes are accompanied by corresponding EEG-derived neurophysiological alterations, exploring longitudinal brain–behavior associations. **Methods**: Fifty participants completed psychological assessments at baseline, 6 months, and 12 months using validated scales (BDI-II, DASS-21, EMAS). A subcohort of 25 participants also underwent EEG recordings with a portable Muse device at the same time points. Longitudinal changes were analyzed using linear mixed-effect models, and exploratory brain–behavior associations were assessed with change-score analyses and Spearman’s correlations with false discovery rate correction. **Results**: Across the full cohort (*n* = 50), psychological outcomes showed longitudinal improvements over 12 months, with reductions in BDI-21, DASS-21 depression, anxiety, and stress subscales, and EMAS-State scores (all *p* < 0.001; linear mixed-effect models). In the EEG subcohort (*n* = 25), longitudinal analyses showed increased alpha power and reduced beta and gamma power in frontal and temporoparietal regions (pFDR < 0.05), along with a modest decrease in delta power at 12 months, while theta power remained stable. Exploratory analyses showed non-significant trends in the hypothesized directions that did not remain statistically significant after correction for multiple comparisons (e.g., Δalpha vs. Δstate anxiety: ρ ≈ −0.44; Δbeta vs. Δdepression: ρ ≈ 0.43) or after FDR correction. **Conclusions**: The mindfulness- and gratitude-based intervention was associated with sustained improvements in psychological outcomes and suggests accompanying dynamic modulation of neurophysiology. EEG appears to reflect time-dependent neural adaptation rather than a static predictor of treatment response.

## 1. Introduction

Anxiety and depressive disorders are among the most prevalent and disabling mental health conditions globally, with chronic perceived stress contributing to disease burden. According to the Global Burden of Disease (GBD) 2021 study, the global prevalence in 2021 was 359.2 million for anxiety disorders and 332.4 million for depressive disorders [1,2]. These conditions are associated with reductions in quality of life, functional impairment, and increased healthcare utilization [3,4]. Cognitive Behavioral Therapy (CBT) demonstrates comparable efficacy to pharmacotherapy in many anxiety and depressive disorders, particularly for mild to moderate symptom severity, with clinically meaningful effect sizes [5,6].

Mindfulness-based interventions (MBIs), acceptance and commitment therapy, nature-based health interventions, and digital self-help modalities demonstrate significant short-term benefits for anxiety, depression, and stress [6,7]. High-quality clinical evidence shows that MBIs, such as Mindfulness-Based Stress Reduction (MBSR) and Mindfulness-Based Cognitive Therapy (MBCT), produce statistically significant reductions in anxiety, depressive symptoms, and perceived stress in adults [6,8,9]. Neuropsychological factors proposed to underlie these effects include enhanced emotion regulation, as evidenced by increased connectivity between prefrontal cortical regions and the amygdala, leading to reduced limbic reactivity and improved top-down control of emotional responses [10,11,12]. However, while these effects are well established at the psychological level, their long-term neurophysiological correlates remain insufficiently characterized, particularly in longitudinal settings.

The Gratitude Touch is an exploratory somatic component conceptualized as a body-based emotional anchoring technique grounded in conscious tactile awareness. It aligns with established somatic approaches that integrate intentional touch into mindfulness-based interventions. Theoretical foundations for such techniques are supported by affective neuroscience and psychophysiology, which demonstrate that intentional tactile stimulation, particularly affective touch activating C-tactile afferents, has been proposed to modulate autonomic nervous system activity, potentially promoting parasympathetic activation, increasing vagal tone, and attenuating stress responses [13]. Such mechanisms have been proposed in the literature; however, their empirical validation, particularly in combination with mindfulness-based interventions, remains limited in neuroscience and psychophysiology, although in the present study, physiological effects are examined indirectly through EEG-derived markers [14,15]. In the present study, these autonomic-related mechanisms are not directly measured but are instead assessed indirectly through EEG-derived neurophysiological markers. However, its independent contribution when combined with mindfulness-based interventions remains to be established.

Neurophysiological biomarkers, particularly EEG spectral power and event-related measures, are increasingly utilized to objectively assess the effects of psychological interventions such as MBIs and CBTs. EEG provides quantifiable indices of brain activity that can elucidate mechanisms underlying changes in arousal, attention, and emotion regulation [16,17,18]. Portable EEG devices, such as the Muse InteraXon^®^ headband, Muse™ headband (InteraXon Inc., Toronto, ON, Canada) have enabled longitudinal and ecologically valid monitoring of neurophysiological changes during psychological interventions. Muse InteraXon^®^ is used in research and has demonstrated sensitivity to changes in the alpha, beta, and theta bands relevant to psychological states, with device metrics correlating with self-reported mindfulness and attentional control [19,20,21]. Validation studies indicate that while Muse InteraXon^®^ provides acceptable signal quality for spectral power measures, it has limited spatial resolution and is more susceptible to artifacts than research-grade systems [21,22].

Current evidence is limited by a scarcity of longitudinal studies exceeding six months that simultaneously evaluate psychological outcomes and their corresponding neurophysiological correlates. Most available research relies on short-term designs or isolated follow-up points, limiting understanding of the temporal stability and mechanisms underlying brain–behavior relationships. Additionally, there is a marked lack of empirical studies examining the integration of mindfulness with gratitude-based somatic techniques, leaving the potential synergistic effects of combined cognitive and somatic interventions on psychological outcomes and neural activity largely unexplored.

To date, few studies have examined long-term (≥12 months) within-subject associations between validated psychological outcomes and EEG-derived neurophysiological changes using portable devices in naturalistic intervention settings [23,24].

This longitudinal study aims to investigate (i) the extent to which a combined mindfulness and gratitude-based intervention reduces psychological distress (anxiety, depression, and perceived stress), and (ii) whether these changes are accompanied by corresponding neurophysiological alterations measured using a portable EEG device. Specifically, this study examines whether temporal changes in depressive symptoms, anxiety, and perceived stress, assessed using the Beck Depression Inventory-II (BDI-21), the Endler Multidimensional Anxiety Scales (EMAS), and the Depression Anxiety Stress Scales (DASS-21), are accompanied by corresponding longitudinal alterations in EEG spectral activity, across canonical EEG frequency bands, with particular interest in alpha- and beta-related activity previously linked to emotional regulation, across baseline, 6-month, and 12-month assessment points. This extended 12-month intervention with weekly sessions provides an opportunity to examine sustained longitudinal trajectories, addressing a key limitation of prior short-term studies.

The central hypothesis is that longitudinal improvements in psychological symptom severity, as reflected by decreases in BDI-21, EMAS, and DASS-21 scores, will be associated with corresponding EEG spectral changes over time, indicating potential brain–behavior relationships. Specifically, these changes are expected to involve increased alpha power and reduced beta activity, patterns commonly linked to enhanced relaxation, emotional regulation, and reduced stress-related cognitive arousal.

## 2. Materials and Methods

### 2.1. Study Design

This study employs a prospective single-arm, longitudinal interventional design with repeated psychological and neurophysiological assessments over 12 months. Participants undergo a standardized mindfulness-based intervention that incorporates a gratitude-focused somatic component, delivered in weekly sessions. Outcomes are evaluated at predefined time points to capture temporal changes and within-subject trajectories. Given the exploratory and longitudinal nature of this study, no control group was included, and no randomization or blinding procedures were applied. This study is designed to evaluate within-subject temporal associations rather than to establish causal effects.

### 2.2. Participants

This study includes 50 adult female participants aged 25 years or older, recruited from the general population. Eligibility criteria include the ability to participate in weekly sessions over the 12-month study period and provision of written informed consent. Exclusion criteria comprise a history of diagnosed neurological disorders, current substance use disorders, or significant changes in psychotropic medication during the study period, to minimize confounding effects on neurophysiological measurements. All participants are assessed at baseline and followed longitudinally according to the predefined evaluation schedule, enabling within-subject analysis of psychological and EEG changes over time.

All participants completed the scheduled intervention period. Detailed quantitative measures of individual adherence or engagement with the intervention were not systematically recorded.

### 2.3. Intervention

The intervention consists of a 12-month (1 December 2024–1 December 2025) structured MBI delivered through weekly sessions, integrating formal mindfulness exercises with the Gratitude Touch somatic technique. Each session follows a standardized protocol (60 min, individual, live session) and is conducted by trained personnel to ensure consistency across participants and time points (the full protocol is available in the Supplementary Materials). The mindfulness component includes guided practices focused on attention to breathing, bodily sensations, and present-moment awareness, aiming to enhance interoceptive awareness and emotional regulation through non-judgmental observation of internal experiences.

The Gratitude Touch is a structured, body-based gratitude exercise designed to promote emotional anchoring through conscious tactile stimulation and verbalized gratitude. As described in the original intervention protocol, participants are guided to sequentially touch each finger of both hands while verbally articulating specific elements of gratitude and their personal significance. This process integrates tactile grounding, cognitive reflection, and affective engagement, followed by a brief period of bodily observation focused on breathing, heart rate, and perceived emotional state. The technique is conceptually designed to facilitate autonomic self-regulation through combined tactile and cognitive engagement; however, its physiological effects are examined indirectly using EEG-derived markers.

### 2.4. Psychological Assessment Instruments

Psychological outcomes are assessed using validated self-report instruments administered at baseline (T0), 6 months (T1), and 12 months (T2), allowing for longitudinal evaluation of depressive symptoms, anxiety, and perceived stress. All instruments are used in clinical and research settings and have demonstrated robust psychometric properties, including reliability, construct validity, and sensitivity to change over time.

Depressive symptoms are measured using the BDI-21, a 21-item scale assessing the severity of cognitive, affective, and somatic symptoms of depression over the preceding two weeks. Each item is rated on a 4-point Likert scale (0–3), yielding a total score of 0–63, with established cut-offs for minimal, mild, moderate, and severe depression. The BDI-II demonstrates excellent internal consistency (Cronbach’s alpha typically >0.90) and strong convergent validity across clinical and non-clinical populations, including validated Romanian versions used in both research and applied psychological assessment [25].

Anxiety is assessed using the EMAS, which conceptualizes anxiety as a multidimensional construct encompassing both state anxiety (EMAS-S) and trait-related situational appraisal (EMAS-P). The EMAS-S consists of items that capture cognitive and somatic components of situational anxiety. In contrast, EMAS-P evaluates perceived threat across distinct contexts (e.g., social evaluation, physical danger, ambiguity, and daily routines). The EMAS has demonstrated good internal consistency, factorial validity, and cross-cultural applicability, and the Romanian adaptation is well established in both clinical and research contexts, supporting its use for longitudinal anxiety assessment [26].

Perceived stress, anxiety, and depressive symptoms are further evaluated using the Depression Anxiety Stress Scales–21 (DASS-21). This instrument comprises three 7-item subscales assessing depression, anxiety, and stress, rated on a 4-point Likert scale. Subscale scores are summed and multiplied by two to maintain comparability with the original 42-item version, and are interpreted according to standardized severity thresholds. The DASS-21 exhibits strong internal consistency (Cronbach’s alpha generally >0.85 across subscales), good convergent validity, and sensitivity to longitudinal change. Validated Romanian versions have been shown to perform reliably in both general and clinical populations, supporting their suitability for repeated-measures designs [27].

### 2.5. EEG Data Acquisition and Preprocessing

EEG signals were recorded using the Muse™ headband (InteraXon Inc., Toronto, ON, Canada) portable EEG system, with four dry electrodes positioned at TP9, AF7, AF8, and TP10, referenced to frontal ground electrodes. Data were sampled at 256 Hz and exported as time-resolved spectral power values. EEG recordings were obtained at baseline (T0), 6 months (T1), and 12 months (T2), corresponding to the same assessment points as the psychological evaluations for the EEG subcohort. The use of the Muse system was motivated by its portability, ease of use, and feasibility for repeated longitudinal assessments in naturalistic settings, despite its known limitations in spatial resolution compared to research-grade EEG systems.

Preprocessing was performed using the device’s proprietary automated pipeline, which included band-pass filtering (1–45 Hz), line-noise correction, automated artifact detection, and internal signal-quality thresholding. Only epochs meeting predefined quality criteria were retained for analysis. No manual artifact rejection procedures (e.g., ICA) were applied.

Relative spectral power was extracted for five canonical frequency bands: delta (1–4 Hz), theta (4–7 Hz), alpha (8–12 Hz), beta (13–30 Hz), and gamma (30–45 Hz). Spectral values were computed for each electrode and subsequently averaged across homologous electrode pairs to derive regional measures for frontal (AF7/AF8) and temporoparietal (TP9/TP10) regions.

Processed data were exported via the Mind Monitor platform, and subsequent statistical analyses were conducted offline in R (version 4.3.3).

It should be noted that no manual artifact rejection procedures (e.g., independent component analysis) were applied, and preprocessing relied exclusively on the device’s proprietary automated pipeline. While this approach ensures standardization, it may not fully eliminate EMG contamination, particularly in higher-frequency bands such as beta and gamma.

### 2.6. Statistical Analysis

Statistical analyses were performed using Python (version 3.12). Descriptive statistics were calculated to characterize the study sample and to summarize psychological and EEG-derived variables at each assessment point. Continuous variables are reported as mean ± standard deviation or median (interquartile range), as appropriate, based on the distribution assessed using the Shapiro–Wilk test.

Longitudinal changes in psychological outcomes (BDI-21 total score, EMAS-S, EMAS-P, and DASS-21 depression, anxiety, and stress subscales) across repeated measurements (T0, T1, T2) were analyzed using linear mixed-effect models (LMMs) with time as a fixed effect and participant as a random intercept, allowing for within-subject correlation and incomplete follow-up data. Model assumptions were evaluated by inspection of residual distributions. When model assumptions were not met, nonparametric Friedman tests were used in sensitivity analyses, and post hoc pairwise comparisons were performed using Wilcoxon signed-rank tests with a Bonferroni adjustment.

EEG spectral power variables (relative alpha, beta, and theta power) were analyzed at baseline (T0), 6 months (T1), and 12 months (T2). Spectral power values were averaged across homologous electrode pairs to obtain frontal (AF7/AF8) and temporoparietal (TP9/TP10) regional measures. Longitudinal changes in EEG parameters were examined using LMM with time as a fixed effect and participant as a random effect. To control for multiple comparisons in EEG spectral analyses and brain–behavior correlation analyses, *p*-values were adjusted using the Benjamini–Hochberg false discovery rate (FDR) procedure.

Associations between longitudinal changes in psychological outcomes and corresponding EEG parameters were examined using change-score analyses. For each participant, change scores were calculated as the difference between 12-month (T2) and baseline (T0) values for both psychological scales and EEG spectral power measures (Δ = T2 − T0). Within-subject associations between psychological improvement and neurophysiological changes were assessed using Spearman’s rank correlation coefficients, given the non-normal distribution of change scores, with *p*-values adjusted for multiple comparisons using the Benjamini–Hochberg false discovery rate (FDR) procedure. Additionally, exploratory linear regression models were constructed to evaluate whether baseline EEG spectral power predicted the magnitude of psychological improvement over the 12-month follow-up.

All statistical tests were two-tailed, and a *p*-value < 0.05 was considered statistically significant after correction for multiple testing where applicable.

### 2.7. Ethics Approval and Informed Consent

This study is conducted in accordance with the principles of the Declaration of Helsinki. The research protocol is approved by the Scientific Research Ethics Committee of the “Victor Babeș” University of Medicine and Pharmacy, Timișoara, under Ethics Approval No. 119, issued in 2022, revised on 23 March 2026. All participants provide written informed consent before enrollment; they can withdraw at any time, and there is no financial compensation. Participant data are anonymized, securely stored, and handled confidentially throughout the research process to ensure privacy and data protection.

## 3. Results

### 3.1. Sample Characteristics and Data Availability

A total of 50 adult female participants were included in the psychological analyses and completed all questionnaire-based assessments at baseline (T0), 6 months (T1), and 12 months (T2). The mean age of the study sample was 31.6 ± 5.1 years, with a median age of 31 years (interquartile range [IQR]: 8 years), indicating a relatively homogeneous cohort of young adults (Table 1).

All participants provided complete psychological data at each assessment point. This complete longitudinal follow-up strengthens the internal validity of the observed temporal trends in psychological outcomes.

A randomly selected subset of participants (n = 25; 50% of the total cohort) also underwent neurophysiological assessment using the Muse portable EEG system. EEG data were successfully acquired for all participants in this subcohort at baseline, 6 months, and 12 months, yielding complete datasets at each time point.

### 3.2. Longitudinal Changes in Psychological Outcomes

Longitudinal mixed-effect modeling demonstrated significant time-dependent improvements across multiple psychological domains over the 12-month intervention period. Linear mixed-effect models with participant as a random effect revealed a robust main effect of time on depressive symptoms, anxiety, and perceived stress.

Depressive symptom severity, assessed using the BDI-21 total score, showed a significant reduction at both 6 months (β = −4.04, 95% CI −4.57 to −3.51, *p* < 0.001) and 12 months (β = −4.02, 95% CI −4.55 to −3.49, *p* < 0.001) compared with baseline. Similarly, state anxiety, measured by the EMAS-State scale, decreased significantly over time, with marked reductions observed at 6 months (β = −7.32, *p* < 0.001) and sustained at 12 months (β = −7.44, *p* < 0.001).

In contrast, EMAS-Trait scores remained stable across all assessment points, indicating no significant longitudinal change in dispositional anxiety. This pattern is consistent with the conceptual distinction between state-dependent and trait-like anxiety dimensions. Analysis of the DASS-21 subscales revealed significant longitudinal decreases in depression, anxiety, and stress scores. Reductions were evident at both follow-up time points, with the largest effects observed at 6 months and sustained improvements at 12 months (all *p* < 0.001) (Table 2).

**Figure 1:**
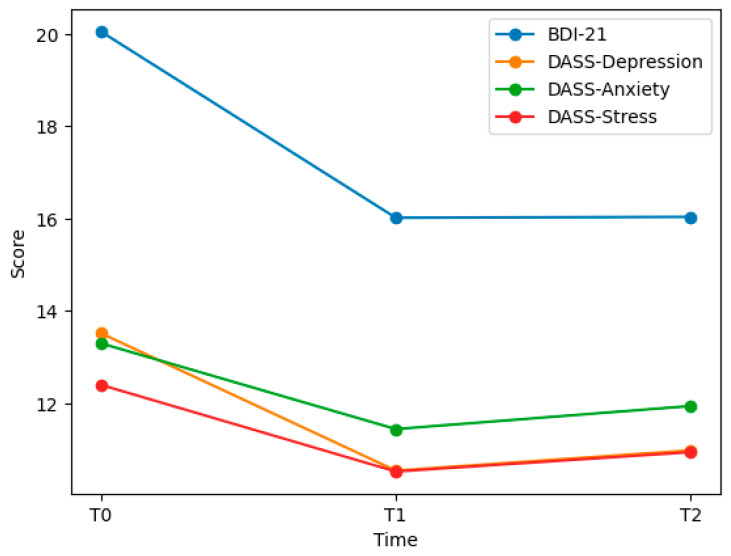
Mean trajectories of psychological outcomes over time.

Sensitivity analyses using non-parametric Friedman tests yielded consistent results, confirming the robustness of the observed longitudinal effects (Figure 1).

### 3.3. Longitudinal Changes in EEG Spectral Power

Within the Muse EEG subcohort (n = 25), longitudinal mixed-effect models demonstrated coherent time-dependent modulation of relative spectral power across canonical frequency bands. Spectral power values were averaged within frontal (AF7/AF8) and temporoparietal (TP9/TP10) regions and analyzed using linear mixed-effect models with time as a fixed effect and participant as a random effect. False discovery rate correction (Benjamini–Hochberg) was applied across all tested time contrasts.

A robust increase in alpha power was observed over time in both regions. Relative alpha increased at 6 months and remained elevated at 12 months compared with baseline (all *p*FDR < 0.001). In parallel, beta power decreased significantly at both follow-up assessments across the frontal and temporoparietal regions (all *p*FDR < 0.001). Gamma power also exhibited significant reductions at 6 and 12 months in both regions (all *p*FDR < 0.001), indicating a consistent shift toward reduced higher-frequency activity over time.

**Figure 2:**
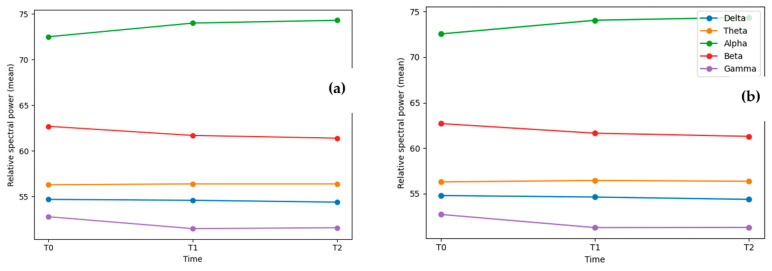
(**a**) Frontal (AF7/AF8) EEG band power trajectories over time; (**b**) Temporoparietal (TP9/TP10) EEG band power trajectories over time.

For lower-frequency bands, delta power showed a small but statistically significant reduction at 12 months (T2 vs. T0) in both frontal and temporoparietal regions (*p*FDR < 0.05), whereas theta power did not demonstrate significant longitudinal changes after correction for multiple testing (all *p*FDR > 0.05) (Table 3, Figure 2a,b).

Overall, the longitudinal EEG profile is characterized by increased alpha power accompanied by decreased beta and gamma power across both cortical regions, with more modest late reductions in delta power and stable theta activity.

### 3.4. Associations Between Psychological Changes and EEG Markers

Exploratory analyses examined the associations between longitudinal psychological improvement and neurophysiological change in the Muse EEG subcohort (n = 25) using change scores from baseline to 12 months. For each participant, change scores were computed for psychological outcomes (Δ = T2 − T0) and for EEG-derived relative spectral power (Δalpha, Δbeta, Δtheta). Spearman’s rank correlations were calculated to assess monotonic associations between psychological and EEG changes, and the false discovery rate (Benjamini–Hochberg) correction was applied to all tested correlations.

Exploratory correlation analyses indicated non-significant trends whereby greater increases in alpha power were associated with larger reductions in state anxiety (ΔEMAS-State vs. Δalpha: ρ = −0.44, *p* = 0.028), while decreases in beta power were associated with greater reductions in depressive symptoms on the DASS-21 depression subscale (ΔDASS-Depression vs. Δbeta: ρ = 0.43, *p* = 0.032). However, these associations were not statistically significant after FDR correction, indicating that the observed brain–behavior relationships should be interpreted as exploratory given the modest sample size.

**Figure 3:**
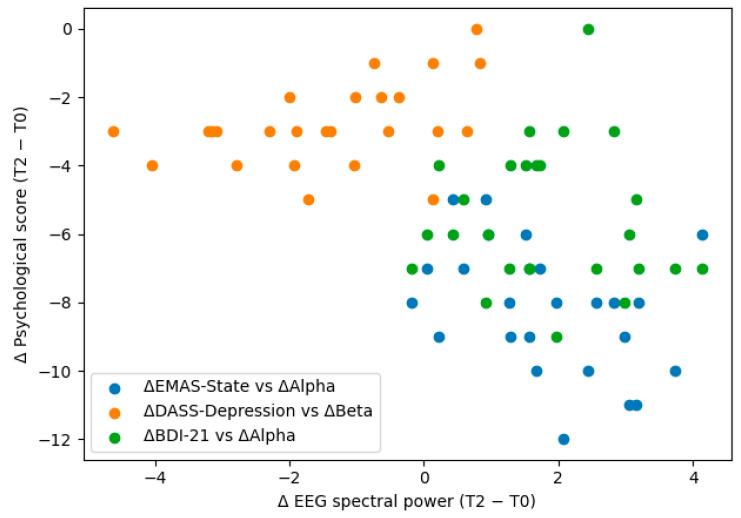
Associations between changes in psychological outcomes and EEG markers.

A confirmatory regression model adjusting for age and baseline state anxiety was consistent with a potential inverse association between Δalpha and ΔEMAS-State (β = −0.76, *p* = 0.022), although not statistically significant after correction, suggesting that increases in alpha activity may accompany improvements in state anxiety over the 12-month period. The findings provide preliminary evidence that psychological improvements may be accompanied by measurable EEG spectral shifts, warranting validation in larger cohorts (Table 4, Figure 3).

### 3.5. Exploratory Predictors of Psychological Improvement

Exploratory linear regression analyses were conducted to investigate whether baseline EEG spectral markers predicted long-term psychological improvement in the Muse EEG subcohort (n = 25). Change scores from baseline to 12 months (Δ = T2 − T0) for depressive symptoms (BDI-21 total and DASS-21 depression subscale) were used as outcomes, with baseline alpha and theta relative spectral power as predictors and age as a covariate.

Baseline alpha and theta power were not significant predictors of change in BDI-21 scores over the 12-month period (all *p* > 0.25). Similarly, neither alpha nor theta power at baseline significantly predicted change in DASS-21 depression scores. Age showed a trend toward association with ΔDASS-21 depression (β = 0.089, *p* = 0.067), although this did not reach conventional statistical significance (Table 5).

These exploratory analyses suggest that baseline EEG spectral power alone does not strongly predict the magnitude of subsequent psychological improvement in this sample. The findings support the interpretation that longitudinal changes in EEG activity, rather than baseline levels, may be more closely linked to clinical improvement.

## 4. Discussion

### 4.1. Summary of Key Findings and Clinical Meaning

This long-term study indicates substantial and sustained improvements over time in psychological health over 12 months. Reductions in depressive symptoms, anxiety, and perceived stress were detected using various validated tools, such as BDI-21, DASS-21 subscales, and EMAS-State. These positive changes were consistent across multiple assessments and were not affected by participant dropout. Overall, the results suggest meaningful longitudinal changes that may reflect potential clinical relevance.

Consistent longitudinal changes in EEG spectral activity were observed within the Muse subcohort. There was an increase in alpha power and a decrease in beta and gamma power across both frontal and temporoparietal regions, with a slight late reduction in delta power, while theta activity remained unchanged. Exploratory analysis indicated that greater psychological improvements tended to coincide with larger EEG spectral changes; however, these brain–behavior links did not remain significant after multiple-comparison correction and should be viewed with caution. Baseline EEG measures did not reliably predict future psychological improvement, suggesting that EEG functions more as a dynamic neural correlate of change than as a static predictor of treatment response. Overall, the results suggest a measurable clinical association with the intervention, indicate that its benefits are linked to adaptive, time-dependent neurophysiological changes, and further support the notion that EEG measures in this context reflect dynamic longitudinal changes rather than baseline predictive biomarkers.

### 4.2. Psychological Outcomes over 12 Months in Context

The observed reductions in depressive symptoms, anxiety, and perceived stress are broadly consistent with the existing mindfulness literature. Numerous studies have reported beneficial effects of mindfulness-based interventions on psychological distress, particularly for depression and anxiety, as measured by instruments such as the BDI and DASS-21. Meta-analyses and reviews indicate that mindfulness practices are generally associated with small-to-moderate improvements in psychological outcomes, although effect sizes vary substantially across populations, intervention formats, and follow-up durations [28,29,30].

Importantly, the magnitude and persistence of psychological improvement observed in the present study are comparable to those reported in a limited number of longitudinal investigations. Low-intensity mindfulness interventions with extended follow-up periods have demonstrated sustained reductions in distress at 6 and 12 months, particularly when ongoing practice or booster components are incorporated. Similarly, randomized and non-randomized trials have reported significant decreases in DASS-21 subscale scores following mindfulness-based programs, although most such studies have limited short-term follow-up to 8–12 weeks or focus on specific clinical or occupational groups [31,32,33].

In contrast, other reviews have highlighted heterogeneous or inconsistent psychological effects of mindfulness interventions, with some studies reporting minimal or non-significant changes, depending on baseline symptom severity, comparator conditions, and adherence levels. Against this mixed background, the present findings are strengthened by complete longitudinal follow-up, repeated assessments over 12 months, and a zero dropout rate. At the same time, the absence of a randomized control group limits causal inference and warrants cautious interpretation [34]. Together, these considerations suggest that the psychological improvements observed here are clinically meaningful and aligned with a subset of the literature, while also reflecting the broader variability reported across mindfulness research.

### 4.3. Longitudinal EEG Spectral Changes: Interpretation and Comparison

The EEG analysis over 12 months showed a clear, consistent spectral change pattern, with most changes emerging by 6 months and remaining relatively stable by 12 months. Relative alpha power increased, while beta and gamma powers decreased in both frontal and temporoparietal areas. Delta power decreased modestly but significantly at 12 months, while theta activity remained stable throughout. Similar patterns across regions indicate a widespread rather than localized neurophysiological change. This temporal pattern mirrors the trajectory observed in psychological outcomes, suggesting an early neurophysiological response followed by longitudinal stabilization.

From a neurophysiological perspective, increased alpha activity is commonly interpreted as reflecting enhanced internalized attention, relaxation, and emotional regulation. Concurrent reductions in beta and gamma power may indicate decreased hyperarousal; however, these findings should be interpreted with caution, as high-frequency EEG bands are particularly susceptible to EMG artifacts, including facial muscle activity and jaw tension. The late reduction in delta power could reflect the gradual normalization of slower oscillatory components associated with vigilance or fatigue. The stability of theta activity suggests that not all frequency bands are equally sensitive to long-term contemplative practice, underscoring the specificity of the observed effects [35,36].

Given the absence of manual artifact rejection procedures, it is not possible to fully exclude the contribution of non-neural signals to the observed longitudinal changes in beta and gamma activity. Therefore, these findings should be considered exploratory and interpreted with caution.

These findings are largely consistent with prior EEG research on meditation and mindfulness. The classic “states and traits” framework describes alpha enhancement and beta attenuation as core electrophysiological features of meditative practice [23]. A systematic review further reports that mindfulness is most consistently associated with increased alpha power, while changes in theta, beta, delta, and gamma are more variable across studies [37]. Longitudinal studies of contemplative training have shown that EEG spectral changes can emerge over time, supporting the plausibility of the present findings. Reductions in resting-state beta power have also been described in longitudinal EEG studies as reflecting adaptive baseline shifts (“shifting baselines”) [38,39,40]. In contrast, some studies report increased gamma activity during brief meditation sessions, likely reflecting transient-state effects [37,41,42]. The longitudinal decrease in gamma observed here may therefore reflect differences in measurement context, or practice-related trait adaptation, consistent with emerging evidence that gamma dynamics depend strongly on task demands and contemplative style. Taken together, these findings support a model of early EEG adaptation followed by sustained neurophysiological stability over time, consistent with longitudinal patterns reported in contemplative training studies.

### 4.4. Brain–Behavior Coupling: Why Signals Are Suggestive but Exploratory

Changes in brain–behavior relationships were analyzed using change-score comparisons between baseline and 12-month assessments (Δ = T2 − T0). The relationship between shifts in psychological measures and EEG spectral power was explored using Spearman’s rank correlation, with FDR correction for multiple comparisons. The results indicated a trend where increased alpha power was linked to decreased state anxiety, and reduced beta power was linked to improved depressive symptoms. However, these trends did not reach statistical significance after FDR correction; so, they remain preliminary observations.

Several factors likely contributed to the limited statistical robustness of these findings. The EEG subcohort was modest in size (n = 25), multiple brain–behavior associations were tested, and the use of a consumer-grade portable EEG device introduces additional signal variability, despite prior validation of the Muse system for research applications [43]. Importantly, randomized trials have demonstrated the feasibility of using Muse for home-based mindfulness interventions, with detectable psychological and neurophysiological effects in selected populations [20]. Taken together, our findings provide preliminary evidence of measurable neurophysiological correlates of psychological improvement, warranting replication in larger, controlled cohorts.

Importantly, none of the observed brain–behavior associations remained statistically significant after correction for multiple comparisons. Therefore, these findings should be interpreted strictly as exploratory and hypothesis-generating, rather than as evidence of robust neurophysiological relationships. The limited sample size and multiple testing burden further reduce statistical power, increasing the likelihood of type II error.

None of the observed brain–behavior associations remained statistically significant after correction for multiple comparisons. Therefore, these findings should be interpreted strictly as exploratory trends rather than evidence of measurable neurophysiological relationships.

### 4.5. Strengths, Limitations, and Future Directions

This study boasts several key strengths. It features repeated psychological assessments paired with longitudinal EEG measurements over a 12-month span, ensuring complete follow-up and no participant dropouts, and weekly sessions, enabling evaluation of sustained within-subject changes over time that are rarely captured in shorter behavioral intervention studies. Combining validated psychological tools with portable EEG recordings allows for ecologically valid, real-world tracking of clinical outcomes and neurophysiological shifts. Additionally, the use of longitudinal modeling techniques and adjustments for multiple testing enhances the methodological robustness of the analysis.

Several limitations should be acknowledged. The EEG subcohort was relatively small (n = 25), reducing the statistical power for brain–behavior analyses and limiting the generalizability of the findings. The lack of a randomized control group limits the ability to draw causal conclusions about the intervention’s effects. Accordingly, this study should be interpreted as exploratory, focusing on within-subject longitudinal patterns rather than causal inference. This design reflects the preliminary nature of this investigation and supports hypothesis generation rather than definitive conclusions about efficacy. This limited sample size may also have contributed to the lack of statistically significant brain–behavior associations after correction for multiple comparisons.

Importantly, the study design does not allow for the disentangling of the specific contribution of the Gratitude Touch component from mindfulness training alone. As a result, the independent effect of the somatic component cannot be determined, and its specific contribution remains unknown.

Additionally, using a consumer-grade EEG device comes with inherent limitations in signal resolution and increased susceptibility to artifacts, even though it has been validated for research use.

In particular, the Muse system relies on a limited number of frontal and temporoparietal electrodes, which restricts spatial resolution and reduces the reliability of detailed regional neurophysiological interpretations compared to research-grade EEG systems. The interpretation of high-frequency EEG findings (beta and gamma bands) is limited by the lack of advanced artifact-rejection techniques, as these bands are particularly vulnerable to EMG contamination.

Furthermore, EEG preprocessing relied on automated artifact rejection without manual cross-validation, which may increase the risk of residual artifacts. In addition, hemispheric differences were not analyzed, as spectral power values were averaged across homologous electrode pairs, a methodological choice driven by the limited sample size of the EEG subcohort. Another limitation is the lack of detailed quantitative assessment of participant adherence and individual engagement with the intervention. Although all participants completed this study, variability in the extent to which the intervention was actively practiced may have influenced the observed outcomes and introduces uncertainty in the interpretation of the results.

Therefore, it is not possible to determine the extent to which the observed psychological improvements are specifically attributable to the intervention rather than to non-specific factors such as time effects or expectancy.

Future studies should strive to replicate these results in larger, controlled groups with preregistered hypotheses and employ controlled or dismantling designs to directly compare mindfulness-only interventions with combined approaches to determine the independent contribution of the Gratitude Touch component. Using randomized designs, incorporating additional physiological markers such as heart rate variability, and conducting more detailed EEG analyses could aid in understanding the mechanisms behind psychological improvements. These efforts are essential to assess whether EEG-based measures can reliably predict long-term responses to mindfulness-based interventions. In particular, future research should employ controlled or dismantling study designs to directly compare mindfulness-only interventions with combined mindfulness-and-Gratitude Touch approaches to determine the specific added value of the somatic component. Also, advanced preprocessing techniques (artifact correction) and multimodal validation approaches should be incorporated to improve the reliability of high-frequency EEG measures.

## 5. Conclusions

This 12-month longitudinal study demonstrates that a mindfulness- and gratitude-based intervention is associated with significant and sustained improvements in psychological well-being, including lower levels of depression, anxiety, and perceived stress. These mental health benefits were mirrored by consistent long-term changes in EEG spectral activity, showing increased alpha power alongside decreased beta and gamma power in frontal and temporoparietal areas. While the brain–behavior links were exploratory and did not remain significant after multiple-comparison correction, non-significant trends in the expected directions were observed; however, these do not provide evidence of measurable neurophysiological markers and should be interpreted as exploratory. Overall, the results suggest potential clinical relevance of the observed changes and suggest that Muse EEG captures dynamic, ongoing neural changes rather than serving as a static predictor of treatment success.

## Figures and Tables

**Table 1:** Sample characteristics.

Characteristic	Full Cohort (Psychological Assessments)	EEG Subcohort (Muse)
Number of participants, n	50	25
Age, years	31.6 ± 5.1	31.4 ± 4.3
Age, median [IQR]	31 [8]	31 [8]
Psychological assessments completed		
– Baseline (T0), n (%)	50 (100%)	25 (100%)
– 6 months (T1), n (%)	50 (100%)	25 (100%)
– 12 months (T2), n (%)	50 (100%)	25 (100%)
EEG assessments completed	—	
– Baseline (T0), n (%)	—	25 (100%)
– 6 months (T1), n (%)	—	25 (100%)
– 12 months (T2), n (%)	—	25 (100%)
Dropout rate at 6 months	0%	0%
Dropout rate at 12 months	0%	0%

**Table 2:** Longitudinal mixed-effect models for psychological outcomes.

Outcome	Time Contrast	β (Estimate)	95% CI	*p*-Value *
BDI-21	T1 vs. T0	−4.04	−4.57 to −3.51	<0.001
	T2 vs. T0	−4.02	−4.55 to −3.49	<0.001
EMAS-State	T1 vs. T0	−7.32	−7.79 to −6.85	<0.001
	T2 vs. T0	−7.44	−7.91 to −6.97	<0.001
EMAS-Trait	T1 vs. T0	0.00	—	1.00
	T2 vs. T0	0.00	—	1.00
DASS-Depression	T1 vs. T0	−2.98	−3.29 to −2.68	<0.001
	T2 vs. T0	−2.54	−2.85 to −2.24	<0.001
DASS-Anxiety	T1 vs. T0	−1.86	−2.13 to −1.59	<0.001
	T2 vs. T0	−1.36	−1.63 to −1.09	<0.001
DASS-Stress	T1 vs. T0	−1.88	−2.23 to −1.53	<0.001
	T2 vs. T0	−1.46	−1.81 to −1.11	<0.001

* Friedman tests.

**Table 3:** Longitudinal changes in EEG spectral power in the Muse subcohort (LMM + FDR).

Region	EEG Band	Time Contrast	β (Estimate)	95% CI	*p*-Value	*p*-Value (FDR)
Frontal	Alpha	T1 vs. T0	1.519	1.013 to 2.025	<0.001	<0.001
Frontal	Alpha	T2 vs. T0	1.834	1.328 to 2.340	<0.001	<0.001
Temporoparietal	Alpha	T1 vs. T0	1.500	0.990 to 2.011	<0.001	<0.001
Temporoparietal	Alpha	T2 vs. T0	1.819	1.308 to 2.329	<0.001	<0.001
Frontal	Beta	T1 vs. T0	−1.061	−1.611 to −0.511	<0.001	<0.001
Frontal	Beta	T2 vs. T0	−1.417	−1.967 to −0.867	<0.001	<0.001
Temporoparietal	Beta	T1 vs. T0	−1.055	−1.604 to −0.505	<0.001	<0.001
Temporoparietal	Beta	T2 vs. T0	−1.402	−1.952 to −0.853	<0.001	<0.001
Frontal	Gamma	T1 vs.T0	−1.444	−1.909 to −0.980	<0.001	<0.001
Frontal	Gamma	T2 vs. T0	−1.429	−1.893 to −0.965	<0.001	<0.001
Temporoparietal	Gamma	T1 vs. T0	−1.444	−1.909 to −0.980	<0.001	<0.001
Temporoparietal	Gamma	T2 vs. T0	−1.424	−1.889 to −0.960	<0.001	<0.001
Frontal	Delta	T1 vs. T0	−0.166	−0.545 to 0.213	0.390	0.665
Frontal	Delta	T2 vs. T0	−0.418	−0.791 to −0.044	0.028	0.041
Temporoparietal	Delta	T1 vs. T0	−0.168	−0.546 to 0.210	0.385	0.665
Temporoparietal	Delta	T2 vs. T0	−0.425	−0.799 to −0.052	0.026	0.039
Frontal	Theta	T1 vs. T0	0.143	−0.330 to 0.616	0.554	0.665
Frontal	Theta	T2 vs. T0	0.057	−0.417 to 0.530	0.814	0.814
Temporoparietal	Theta	T1 vs. T0	0.159	−0.312 to 0.630	0.508	0.665
Temporoparietal	Theta	T2 vs. T0	0.069	−0.402 to 0.541	0.773	0.814

**Table 4:** Spearman’s correlations between change in psychological outcomes and change in EEG parameters (T2 − T0) in the Muse EEG subcohort (n = 25).

Psychological Change (Δ = T2 − T0)	EEG Change (Δ = T2 − T0)	Spearman’s ρ	*p*-Value	*p*-Value (FDR)
ΔBDI-21 total	ΔAlpha	−0.087	0.6808	0.8222
ΔBDI-21 total	ΔBeta	0.071	0.7365	0.8222
ΔBDI-21 total	ΔTheta	0.062	0.7681	0.8222
ΔEMAS-State	ΔAlpha	−0.438	0.0285	0.2394
ΔEMAS-State	ΔBeta	−0.016	0.9398	0.9398
ΔEMAS-State	ΔTheta	−0.329	0.1086	0.4072
ΔDASS-Depression	ΔAlpha	−0.094	0.6558	0.8222
ΔDASS-Depression	ΔBeta	0.430	0.0319	0.2394
ΔDASS-Depression	ΔTheta	0.239	0.2494	0.5344
ΔDASS-Anxiety	ΔAlpha	−0.178	0.3978	0.7293
ΔDASS-Anxiety	ΔBeta	0.298	0.1477	0.4431
ΔDASS-Anxiety	ΔTheta	0.347	0.0895	0.4072
ΔDASS-Stress	ΔAlpha	−0.051	0.8087	0.9104
ΔDASS-Stress	ΔBeta	0.171	0.4143	0.7767
ΔDASS-Stress	ΔTheta	0.274	0.1843	0.4609

ΔEMAS-Trait was constant across participants (no variability), and correlations were not defined.

**Table 5:** Exploratory linear regression models predicting psychological improvement.

Outcome	Predictor	β	SE	*p*-Value
ΔBDI-21	Alpha (baseline)	0.069	0.469	0.884
ΔBDI-21	Theta (baseline)	0.553	0.497	0.278
ΔBDI-21	Age	−0.011	0.081	0.897
ΔDASS-Depression	Alpha (baseline)	0.007	0.265	0.980
ΔDASS-Depression	Theta (baseline)	0.157	0.281	0.582
ΔDASS-Depression	Age	0.089	0.046	0.067

β coefficients represent unstandardized estimates. Δ scores represent change from baseline to 12 months (T2 − T0).

## Data Availability

The data supporting this study are available from the corresponding author upon request. However, for ethical reasons, they are not publicly accessible.

## Supplementary Materials

The following supporting information can be downloaded at: https://www.mdpi.com/article/10.3390/brainsci16040425/s1, Supplementary File: Intervention and EEG Monitoring Protocol.

## Abbreviations

The following abbreviations are used in this manuscript:

AF7, AF8	Frontal EEG electrodes (Muse system)
BDI-II	Beck Depression Inventory, Second Edition
CBT	Cognitive Behavioral Therapy
DASS-21	Depression, Anxiety, and Stress Scale, 21-item version
EEG	Electroencephalography
EMAS	Endler Multidimensional Anxiety Scales
EMAS-S	Endler Multidimensional Anxiety Scales—State
EMAS-P	Endler Multidimensional Anxiety Scales—Perception
FDR	False discovery rate
IQR	Interquartile Range
LMM	Linear Mixed-effect Model
MBCT	Mindfulness-Based Cognitive Therapy
MBI	Mindfulness-Based Intervention
MBSR	Mindfulness-Based Stress Reduction
TP9, TP10	Temporoparietal EEG electrodes (Muse system)
