# Supplementary material for: Longitudinal Effects of Mindfulness Combined with Gratitude Touch on Anxiety, Depression, and Stress: A 12-Month Portable EEG-Based Study
Source: Brain Sci. 2026 Apr 18;16(4):425. doi: 10.3390/brainsci16040425 (PMC13114296; doi:10.3390/brainsci16040425)
Supplement: Supplementary file 1 [file brainsci-16-00425-s001.zip › brainsci-4247430-supplementary.pdf]

brain sciences

Intervention and EEG Monitoring Protocol

Research Project – Structured Mindfulness-Based Intervention Integrating The Gratitude Touch

---

1. Study Design Overview

This intervention represents Phase II of a three-phase longitudinal doctoral research project examining the psychological and neurophysiological effects of a structured mindfulness-based intervention (MBI) integrating a novel somatic gratitude technique (The Gratitude Touch).

Study Phases

- **T0 (Baseline):** Psychological assessment + EEG recording
- **Phase II:** 3-month structured intervention (12 weekly sessions)
- **T3 (6 months post-baseline):** Psychological reassessment
- **T4 (12 months post-baseline):** Final reassessment

Sample

- Total sample: 50 female participants (ages 25–45)
- EEG subgroup: 25 participants

Psychological Measures (T0, T3, T4)

- Depression Anxiety Stress Scales (DASS-21)
- Endler Multidimensional Anxiety Scales (EMAS – state and trait)
- Beck Depression Inventory-II (BDI-II)

Expected outcomes include reductions in stress, anxiety, and depressive symptomatology at 6- and 12-month follow-ups.

---

2. Intervention Structure

The intervention consisted of a structured mindfulness-based program delivered over 3 months.

- 12 individual sessions
- Weekly frequency
- 60 minutes per session
- Delivered face-to-face in a private clinical setting
- Conducted by the same licensed clinical psychologist
- Standardized structure across all participants

The protocol integrates:

1. Formal mindfulness training
2. The Gratitude Touch somatic technique
3. EEG-monitored session (subgroup)

---

3. Standardized Session Format (60 Minutes)

Each session followed the same structure to ensure procedural consistency.

1. Clinical Check-In (5 minutes)

- Subjective emotional state rating (0–10 scale)
- Stress/anxiety rating
- Review of home practice adherence

2. Psychoeducation (10 minutes)

Brief structured explanation covering:

- Stress physiology
- Emotional regulation mechanisms
- Cognitive distortions (CBT framework)
- Interoceptive awareness

Purpose: strengthen cognitive insight into emotional processes.

3. Formal Mindfulness Practice (15 minutes)

brain sciences

Guided exercises including:

- Focused breathing (5 min)
- Body scan (7 min)
- Open monitoring of thoughts/emotions (3 min)

Core principles:

- Non-judgmental awareness
- Decentering
- Emotional labeling

4. The Gratitude Touch Technique (15 minutes)

Structured somatic-cognitive intervention involving:

- Upright seated posture, feet grounded
- Sequential bilateral finger stimulation
- For each finger:
    - Verbal articulation of one gratitude element
    - Brief reflection on personal significance
    - 10–15 seconds emotional anchoring

Standardized gratitude domains:

- Physical health
- Significant relationships
- Personal strengths
- Meaningful past experiences
- Future-oriented hope

The technique combines tactile grounding, cognitive reflection, and affective engagement.

5. Integrated Interoceptive Monitoring (10 minutes)

Silent observation of:

- Breathing rhythm
- Perceived heart rate
- Muscle tension
- Emotional tone

Neutral prompts used to minimize expectancy effects.

6. Integration and Homework Assignment (5 minutes)

- Reflection on emotional shifts
- Assignment of home practice (10–15 minutes, minimum 3 times/week)

---

4. EEG Recording Protocol (Muse Device)

EEG recordings were performed in a subgroup of 25 participants using the Muse headband (portable 4-channel EEG system developed by InteraXon Inc.)

Recordings were conducted in a quiet clinical environment under standardized conditions.

EEG Recording Structure (15 minutes total)

1. **Resting Baseline (3 minutes)**
    - Eyes closed
    - No instruction
    - Spontaneous neural activity
2. **Guided Breathing (5 minutes)**
    - Structured diaphragmatic breathing
    - No gratitude component
3. **Gratitude Touch Execution (5 minutes)**
    - Full bilateral tactile protocol
    - Verbalized gratitude statements
4. **Post-Intervention Rest (2 minutes)**

brain sciences

- Eyes closed
- No guidance

Time-stamped EEG sessions were distributed across working days between 01.12.2024 – 01.12.2025 to ensure consistency and ecological validity.

---

5. EEG Variables and Analytical Focus

From Muse exports (Mind Monitor format), the following band powers were extracted:

- High Beta (associated with cortical hyperarousal)
- Alpha power (associated with relaxed attentional regulation)

Derived metric:

- Beta/Alpha ratio

Observed Pattern (Preliminary Data Summary)

Across the 25-participant EEG subgroup:

- Reduction in high beta activity during guided breathing and Gratitude Touch phases compared to baseline
- Increase in alpha power during Gratitude Touch
- Sustained alpha elevation during post-intervention rest
- Decrease in beta/alpha ratio post-intervention

These neurophysiological changes were analyzed alongside psychological outcome measures.

---

6. Compliance Criteria

Participants were included in per-protocol analyses if they:

- Attended at least 10 of 12 sessions
- Completed baseline EEG (for subgroup)
- Completed T3 and T4 psychological assessments

Participants with attendance below 60% were excluded from per-protocol analysis but retained in intention-to-treat analysis.

---

7. Conceptual Mechanism of Action

The integrated intervention is designed to influence:

- Interoceptive awareness
- Emotional regulation capacity
- Cognitive decentering
- Autonomic balance (indirectly inferred)
- Positive affect activation

The Gratitude Touch technique combines tactile stimulation with structured cognitive-emotional processing, hypothesized to facilitate self-regulatory mechanisms reflected both in psychometric outcomes and EEG-derived markers.
